# Sustainability and COVID-19: Recycling and Reusing N95’s

**DOI:** 10.12669/pjms.36.6.3342

**Published:** 2020

**Authors:** Maria Sabahat, Muhammad Talha Naeem, Anusha Sultan Meo

**Affiliations:** 1 Dr. Maria Sabahat BSc, MBBS, National University of Medical Sciences (NUMS), Rawalpindi, Pakistan; 2 Dr. Muhammad Talha Naeem BSc, MBBS, National University of Medical Sciences (NUMS), Rawalpindi, Pakistan; 3 Anusha Sultan Meo, 4^th^ Year Medical Student, National University of Medical Sciences (NUMS), Rawalpindi, Pakistan

Ever since the Novel Corona Virus Disease (COVID-19) outbreak originated from a local food market of Wuhan, China on 31^st^ December 2019, the modern world has been facing an ongoing crisis in the form of a global pandemic declared by the World Health Organization (WHO) on 11th March, 2019.^1^

With meteoric escalation of cases amidst this seemingly endless medical emergency, there is a severe shortage of medical supplies in health care facilities around the globe. With no definitive treatment or vaccine available at present, preventative hygiene measures and social distancing have been the only rays of hope in trying to flatten the disease curve.

The N95 surgical mask, a tough yet flexible, non-woven polypropylene fiber FFR (filtering facepiece respirator), forms the cornerstone of personal protective equipment (PPE) worn by healthcare workers to prevent contracting the culprit of this pandemic, SARS-COV2. With the letter “N” standing for “Non-Oil” as a Respirator Rating Letter Class and “95” indicating its 95% efficacy, this mask filters out contaminants that are more than 0.3 microns in particulate size as stated by the Centers for Disease Control and Prevention (CDC.)^2^

With the shortage at hand, safe methods of recycling the N95s have now been sought out. Modern research has investigated the impact of various decontamination methods on filtration efficiency, facepiece fit and the ability to reduce viable virus or bacteria on the FFRs.^3^ The most desirable methods are those that reduce the pathogen burden, maintain the function of the FFR and present no residual chemical hazard.

The Vaporized Hydrogen Peroxide (VHP) involves exposure of the masks to vapors of H202, at a minimum of 50-100ppm 4 and can be used to decontaminate masks in volume, a recommendation that is backed by the U.S. Food and Drug Administration. 5 This method has been assessed by two treatment levels, as revealed by the CDC.^6^ Both the Battelle Report and Bergman et. al. revealed that this technique had a minimal effect to filtration efficacy and demonstrated 99.9999% efficiency in killing bacterial spores. Also, no reduction of filtration performance of ten N95 FFR models was seen while showing a 6-log reduction in *Geobacillus stearothermophilus* spores while preserving the integrity of the masks’ fit for up to three uses, a National Institutes of Health (NIH) study showed. 7-9

The next method evaluated was of Ultraviolet (UV) germicidal radiation of wavelength energy 0.5–950 J/cm^2^. It proved to be an efficient method of disinfection, allowing the masks to be used three times while maintaining performance upto 95-100% according to the CDC, with the duration of exposure depending on the intensity of the UV lamp. However, a drawback of this technique is the lack of killing of all viruses and bacteria due to shadow effects produced by the structure of the FFR. Moreover, precaution needs to be taken to avoid exposure to skin and eyes.

The application of moist heat was also evaluated with conditions of 60°C and 80% relative humidity (RH) where there was negligible degradation in the filtration rendering upto the mark performance of the tested FFRs. Research according to Heimbuch et al. revealed that disinfected FFRs contaminated with H1N1 influenza using moist heat, of 65°C and 85% RH, achieved a minimum of 99.99% reduction in virus.^10^ However, one drawback has been noted in this procedure which is that the different pathogens require different levels of disinfection, hence efficacy of treatment varies.

The above mentioned techniques involve the usage of technology to ensure safe recycling of masks. However, a recent NIH study has revealed that SARS-CoV-2 remains on plastic, stainless steel, and cardboard surfaces, surviving for up to 72 hours .^11^ A new proposed strategy is to issue five respirators to each healthcare worker who will then wear one mask each day and store it in a breathable paper bag at the end of each shift. The order should be repeated with a minimum of five days between each FFR use. This technique is however, only recommended when supplies are not as constrained and five respirators are available for each worker, otherwise FFR decontamination becomes necessary.

On the other hand, treatment procedures that affected the efficacy of the masks, the respirator’s fit and seal hence endangering its main purpose of safety, were deemed unfit. These included autoclave, dry heat, isopropyl alcohol, soap and dry microwave irradiation.

To summarize, the Vaporized Hydrogen Peroxide, Ultraviolet (UV) germicidal radiation, moist heat and the usage of 5 respirators have proven to be the most efficient and safe methods of recycling and should be implemented in health care facilities around the globe to ensure the deliverance of Personal Protective Equipment (PPE) to all healthcare workers as a compulsion.

Despite these new tested techniques that practice sustainability at such unprecedented times of crisis, health care practitioners must also be sure to take all the basic precautionary measures before using the decontaminated masks to ensure full gear protection. This includes washing of hands with soap or using a minimum 60% alcohol sanitizer, the usage of sterile gloves when donning the FFR and cross checking its seal, avoiding contact with the inside of the respirator and lastly ensuring the straps, nose bridge and nose foam material of the mask is fully intact.

The greatest threat to the maintenance of global peace and security for human survival has emerged from this pandemic of corona virus, for which neither individuals nor nations were prepared; a disease whose threat has surpassed the heralds of nuclear war, war on terror and even the climate change.^12^ Therefore, in times of such crisis, only with such well-planned strategical measures will medical practitioners be able to defend themselves and attain their basic right of self-protection while serving patients and fighting as frontline warriors against COVID-19.
